# A T Cell View of the Bone Marrow

**DOI:** 10.3389/fimmu.2016.00184

**Published:** 2016-05-17

**Authors:** Adriana Bonomo, Ana Carolina Monteiro, Triciana Gonçalves-Silva, Eric Cordeiro-Spinetti, Rômulo Gonçalves Galvani, Alex Balduino

**Affiliations:** ^1^Cancer Program (Fio-Cancer), Oswaldo Cruz Foundation, Rio de Janeiro, Brazil; ^2^Laboratory on Thymus Research, Oswaldo Cruz Institute, Oswaldo Cruz Foundation, Rio de Janeiro, Brazil; ^3^Immunology and Inflammation Graduate Program, Paulo de Góes Microbiology Institute, Federal University of Rio de Janeiro, Rio de Janeiro, Brazil; ^4^Cell Biology and Technology Laboratory, Veiga de Almeida University, Rio de Janeiro, Brazil; ^5^Microbiology Graduate Program, Paulo de Góes Microbiology Institute, Federal University of Rio de Janeiro, Rio de Janeiro, Brazil; ^6^Excellion Laboratory, Amil/UnitedHealth Group, Petrópolis, Brazil

**Keywords:** T cells, bone remodeling, hematopoiesis, B cells, osteoclast, osteoblast

## Abstract

The majority of T cells present in the bone marrow (BM) represent an activated/memory phenotype and most of these, if not all, are circulating T cells. Their lodging in the BM keeps them activated, turning the BM microenvironment into a “memory reservoir.” This article will focus on how T cell activation in the BM results in both direct and indirect effects on the hematopoiesis. The hematopoietic stem cell niche will be presented, with its main components and organization, along with the role played by T lymphocytes in basal and pathologic conditions and their effect on the bone remodeling process. Also discussed herein will be how “normal” bone mass peak is achieved only in the presence of an intact adaptive immune system, with T and B cells playing critical roles in this process. Our main hypothesis is that the partnership between T cells and cells of the BM microenvironment orchestrates numerous processes regulating immunity, hematopoiesis, and bone remodeling.

The presence of T cells inside the bone marrow (BM) cavity has been reported mostly for central memory T cells (Tcm). It is suggested that hematopoietic stem cells (HSCs) niches serve as “hubs” for optimal T cell maintenance, as T cells can survive in the absence of antigen, in an environment rich in interleukin-7 (IL-7) and IL-15 (1), and other mediators believed to be important to T cell memory maintenance (2). Naive T cells can also be primed, get activated, and develop into effector cells inside the BM. Altogether, the BM can be seen as a primary lymphoid organ, which supports primary and secondary T cell responses (3). However, in the BM, the hematopoietic and the bone remodeling systems share the same microenvironment, together with T cells, which come from the periphery. These three components are maintained in equilibrium under homeostatic conditions. Nonetheless, when T cells are activated, this equilibrium is disrupted, and the result depends on the class of T cell response and its impact on hematopoiesis and bone remodeling.

The next sections will describe and discuss the interactions of BM T cells with the hematopoietic and bone remodeling systems. The first section starts describing the development and organization of the BM hematopoietic microenvironment, discussing the perivascular and endosteal niches and their players. In the second section, the role of T cell on shaping hematopoiesis and the correlation between the class of the adaptive response and type of innate immunity stimulated by specific T cells is presented as a cooperative ensemble. In the third section, T cell influence on bone remodeling is described, as well as the T cell-mediated interactions occurring either in physiological conditions or in two bone pathologies (arthritis and cancer metastasis). The main molecular axis that controls bone remodeling, RANK–RANK-L–OPG, is shared with T and B cells, and is discussed as the molecular basis for functional interaction between the skeletal and the adaptive immune systems. Finally, in the fourth section, T cell activities on both hematopoietic and bone remodeling systems are presented and the ability of T cells to orchestrate both systems is discussed according to the class of the response.

## Hematopoietic Stem Cell Niche in the BM: Close to the Bone Surface, Not Far from the Blood Vessel

### Establishment of the HSC Niche in the BM

During embryo development, first HSCs emerge in the blood islands in the extra-embryonic mesoderm of the yolk sac (4–6). Classified as a primitive population of HSCs; HSCs differentiate into nucleated erythrocytes and monocyte-like myeloid cells. In a second wave of hematopoiesis, termed definitive hematopoietic, HSCs emerge from the intra-embryonic mesoderm of the para-aortic-splanchnopleura/aorta-gonad-mesonephros (AGM) region. These cells, but not those derived from the yolk sac, are capable of fully reconstituting the BM of a lethally irradiated adult animal for 6 months or longer, indicating their definitive properties (4–8).

**Figure 1:**
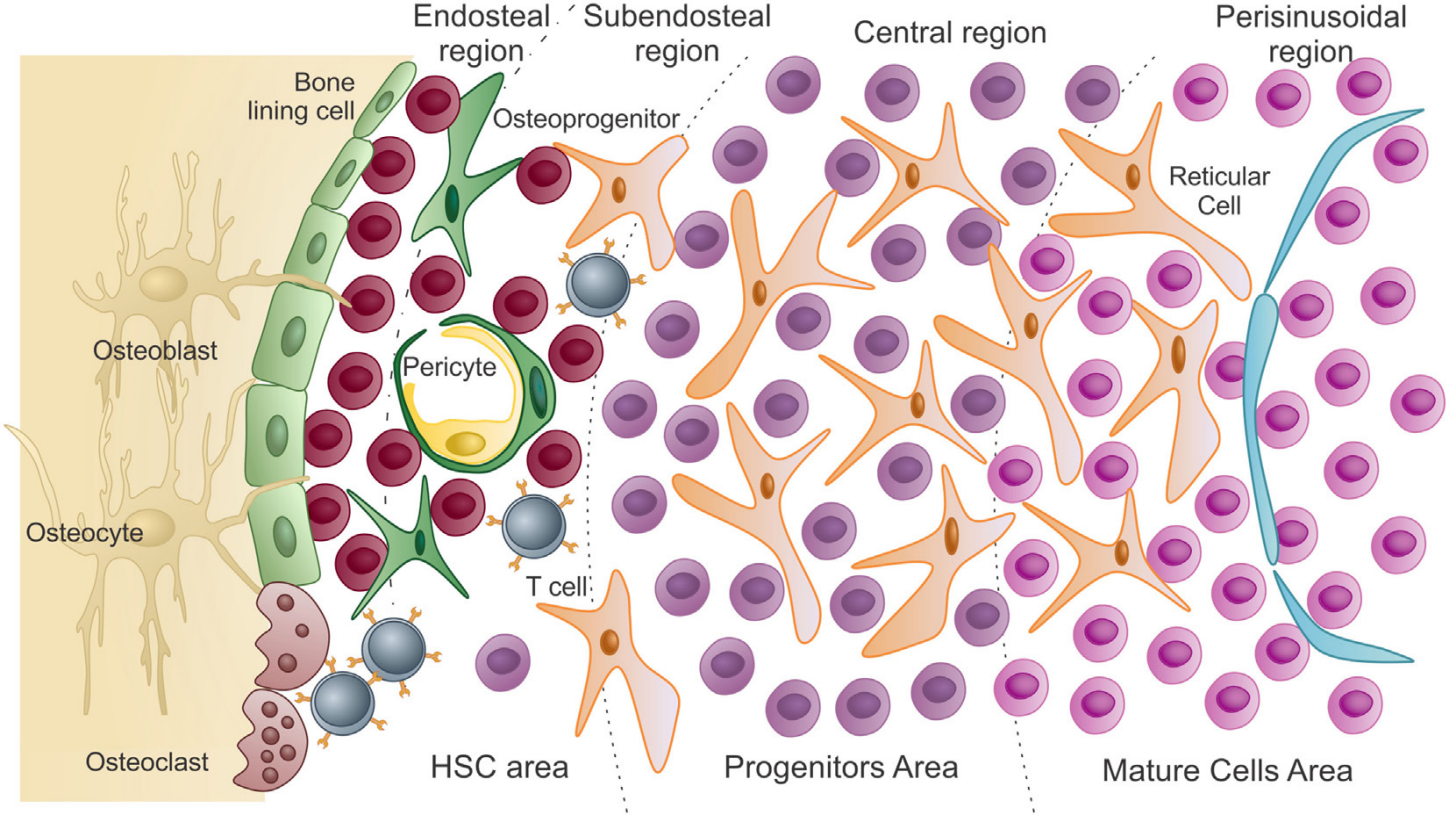
**Schematic presentation of the bone marrow microenvironment under “homeostatic” conditions**. As previously described by Lambertsen and Weiss (15), HSC area, which harbors hematopoietic stem cells and uncommitted progenitors, comprises both endosteal and subendosteal niches. Committed progenitors and differentiated cells are distributed in the central and perisinusoidal niches, respectively. Quiescent hematopoietic stem cells are in close association with endosteal osteoblasts and bone-lining cells. As HSCs exit quiescence to proliferative states, they migrate and colonize the subendosteal perivascular niche, interacting with both endothelial cells and pericytes. Subendosteal sinusoid-derived pericytes serve as source for new osteoprogenitors, which will differentiate into osteoblasts during bone remodeling (adapted from Journal of Cellular Biochemistry, 2014, Cordeiro-Spinetti et al. with permission from John Wiley & Sons).

After blood circulation is established in the embryo, HSCs from both the yolk sac and the AGM region migrate and colonize the fetal liver. The fetal liver microenvironment is responsible for expanding the numbers of HSCs and promoting full commitment of the mesoderm-derived hematopoietic progenitors (9–13). Fetal liver HSC numbers progressively increase, prior to the migration of the hematopoietic system to the recently formed BM cavities. Once established in the BM cavity, most primitive hematopoietic progenitors and stem cells reside in the endosteal and subendosteal regions. Committed progenitors and differentiated cells derived from stem cells will then occupy the central and perisinusoidal regions of the BM cavity, respectively (Figure 1) (14–18).

The BM vasculature is composed of a dense network of blood vessels widely spread within the cavity. Along the bone diaphysis axis, the central artery carries in arterial blood to supply BM cells demand, while, in the same region, the central sinus carries out venous blood and differentiated hematopoietic cells into the blood circulation. Finer arteries and arterioles branch out from the central artery prior to reaching the endosteal surfaces, where they create a web-like structure to provide nutrients to the HSC niche (19–21). Conversely, sinusoidal blood venules cross the BM cavity and coalesce into wider veins as they drain into a central sinus. Approximately, one-fifth of all long-term HSCs in the BM, identified by the expression of CD150, c-Kit, and Sca-1, and absence of lineage markers, are found in close association with endosteal osteoblasts (18, 22–32) The remaining 80% dwell in the abluminal surface of the subendosteal sinusoids, interacting with pericytes and endothelial cells, both contributing to HSC self-renewal (28, 33–35). These data derived from different groups indicate that the niche formed by endosteal osteoblasts and the subendosteal sinusoidal perivascular cells coexist and are referred “quiescent niche” and “proliferative niche,” respectively. *In vitro* and *in vivo* studies suggest that endosteal osteoblasts in the niche stimulate self-renewal and quiescence of the HSC (24, 29, 31, 32, 36, 37). Such that in transgenic animals, increased or decreased numbers of osteoblasts result in increased or decreased numbers of long-term HSC, respectively, without affecting any of the other hematopoietic lineages in the BM (24, 31, 32, 38). *In situ* observations show that most of the HSC population is concentrated in the trabecular bone zone, which also concentrates high numbers of osteoblasts, sinusoids, and CXCL12-positive pericyte-like cells (35, 39). In either case, HSCs are supported by highly expressing CXCL12-positive cells (40). The existence of the two niches remains controversial.

In the BM cavity, the microenvironment organization must match the hematopoietic system differentiation cascade, and each niche composition depends upon quality and number of specific types of stromal cell populations. Stromal cells comprise almost all non-hematopoietic cells found in the BM cavity – osteoblasts, endothelial cells, perivascular cells, mesenchymal cells, and nerve cells – which assemble in an organized network to physically and biologically properly support hematopoiesis (16, 18, 23, 24, 32, 41). Among all stromal cells, mesenchymal population represents around 90%, including pericytes, mesenchymal stem cells (MSCs) and progenitors, perivascular cells, non-perivascular reticular cells, adventitial cells, and all types of myofibroblasts.

### Adaptive Immune Cells in the BM

Translating the spatial organization of niches in conjunction with hematopoietic differentiation, it is conceivable that blood cell production follows a radial pattern within the BM cavity, with most primitive cells residing close to the bone surface and differentiated cells exiting the BM cavity through a central venous sinus (15, 16) (Figure 1). A few differentiated hematopoietic cells leave the BM cavity, circulate in the peripheral blood, differentiate inside other organs as T cells in the thymus, and then return to the BM cavity, migrating to specific niches and playing significant roles as “stromal cells.” As such, T cells participate in a paracrine and juxtacrine signaling to contribute to hematopoiesis. B and T lymphocytes are among those cells with the ability to return to the BM after a quick trip to peripheral blood and lymphoid tissues. After BM cavity entering, memory B-lymphocytes, plasma cells, and T cells concentrate in the perivascular niche of subendosteal sinusoids (Figure 1). They will dwell in close association with CXCL12 highly producing cells, from where they produce and secrete cytokines to help maintain hematopoiesis homeostasis as we know it (23, 42–46).

Recirculating memory B-lymphocytes and active plasma cells secrete considerable levels of IL-6 and TGF-β, contributing to a myelopoietic balance (45, 46). CD4^+^ T cells express CXCR4, which promotes their migration and colonization close to CXCL12 highly producing subendosteal sinusoid perivascular cells. CXCR4 expressing T cells provide substantial assistance to differentiating B-lymphocytes and produce membrane bound and secreted cytokines that regulate myelopoiesis (42, 45, 46). Of note, recirculating B and T cells will colonize those niches identified as HSCs proliferative niche.

### Adipocytes in the BM

Among those cells composing the stromal population, BM adipocytes apparently present the potential to contribute to both local and systemic metabolism (47, 48). BM adipose tissue (MAT) accumulation occurs during aging and other clinical conditions, including osteoporosis, diabetes, and gonadal dysfunction (48, 49), and interventions, such as chemotherapy and radiation (47, 49).

Osteoblasts, hematopoietic-supporting reticular cells, and BM adipocytes derive from a common precursor, named BM MSC, also known as skeletal stem cell or stromal stem cell (47–52). Clinical and histomorphometric studies have shown a correlation between MAT accumulation and decreased bone formation and increased bone loss (48), as hormones, corticoids, and local and peripheral factors might induce mesenchymal progenitors to follow the adipogenic differentiation path at the expense of the osteogenic one. Recent data, however, indicate that the hypothesis of a direct impact of MAT accumulation on skeletal tissue balance disruption is controversial, as adipocytes in the BM may present distinctive phenotypes, resembling either brown or white adipocytes, with potential different interactions with the skeletal tissue (53, 54).

Marrow adipose tissue accumulation and bone loss during aging and other conditions is usually accompanied by a decrease in HSCs number and blood cells production (55–59). In humans, when MAT areas mature in the yellow BM, adipocytes become densely packed and leave no room for active hematopoiesis (48, 60). In humans, yellow BM adipocytes appear at or right before birth, increasing up to the eighth week of age. Larger animals have more yellow BM as compared to smaller animals, which also extends farther into the bone cavities (60–63).

Adipocytes in the red BM do not become packed, but several findings implicate them as negative regulators of hematopoiesis. Adipocyte-derived soluble factors inhibit both B lymphopoiesis and granulopoiesis *in vitro* by blocking committed progenitors differentiation (64–67). When co-cultured with BM adipocytes, HSCs show reduced self-renewal capacity as compared to those co-cultured with other stromal cells (23, 68–71). *In vivo*, BM cavity areas rich in adipocytes most likely hold lower numbers of HSCs and short-term repopulating cells (72). Moreover, wild-type HSCs present enhanced repopulation ability when transplanted into animals with reduced numbers of BM adipocytes (73, 74). It was also observed that the balance between osteoblast and adipocyte differentiation influences hematopoietic recovery after irradiation. Hematopoietic precursor numbers increase when Runx2 expression peaks and PPAR gamma expression decreases, suggesting that osteogenesis but not adipogenesis supports hematopoiesis (73). All data published so far support a negative effect of BM adipocytes in hematopoiesis, even though further investigation is definitely necessary.

In pathological conditions, primary or metastatic tumor cells can grow in the BM. In respect of the niches of tumor cells in the BM, the role played by BM adipocytes is not clear yet. Apparently, BM adipocytes might secrete factors, which will modulate hematopoietic, immune, and tumor cells behavior in the niche. However, it is also very likely that the disruption in the osteoblast/adipocyte differentiation balance results in a new combination of stromal cells and formation of different categories of niches in the BM cavity.

## T Cell in Hematopoiesis: Orchestrating the Peripheral Demand

### T Cell Help Keeps “Normal” Hematopoiesis in the BM

One of the first links between T lymphocytes and hematopoiesis emerges during development, and the relationship persists throughout life (75, 76). *In vitro*, it was shown that T cells could instruct splenic hematopoiesis in response to syngeneic stimulation (77). Also, patients receiving T cell depleted allogeneic BM transplantation have higher rates of graft failure (78, 79). Nonetheless, when CD3^+^ cells were co-administered with allogeneic BM stem cells, improvement and optimization of hematopoietic cells engraftment is observed (80).

**Figure 2:**
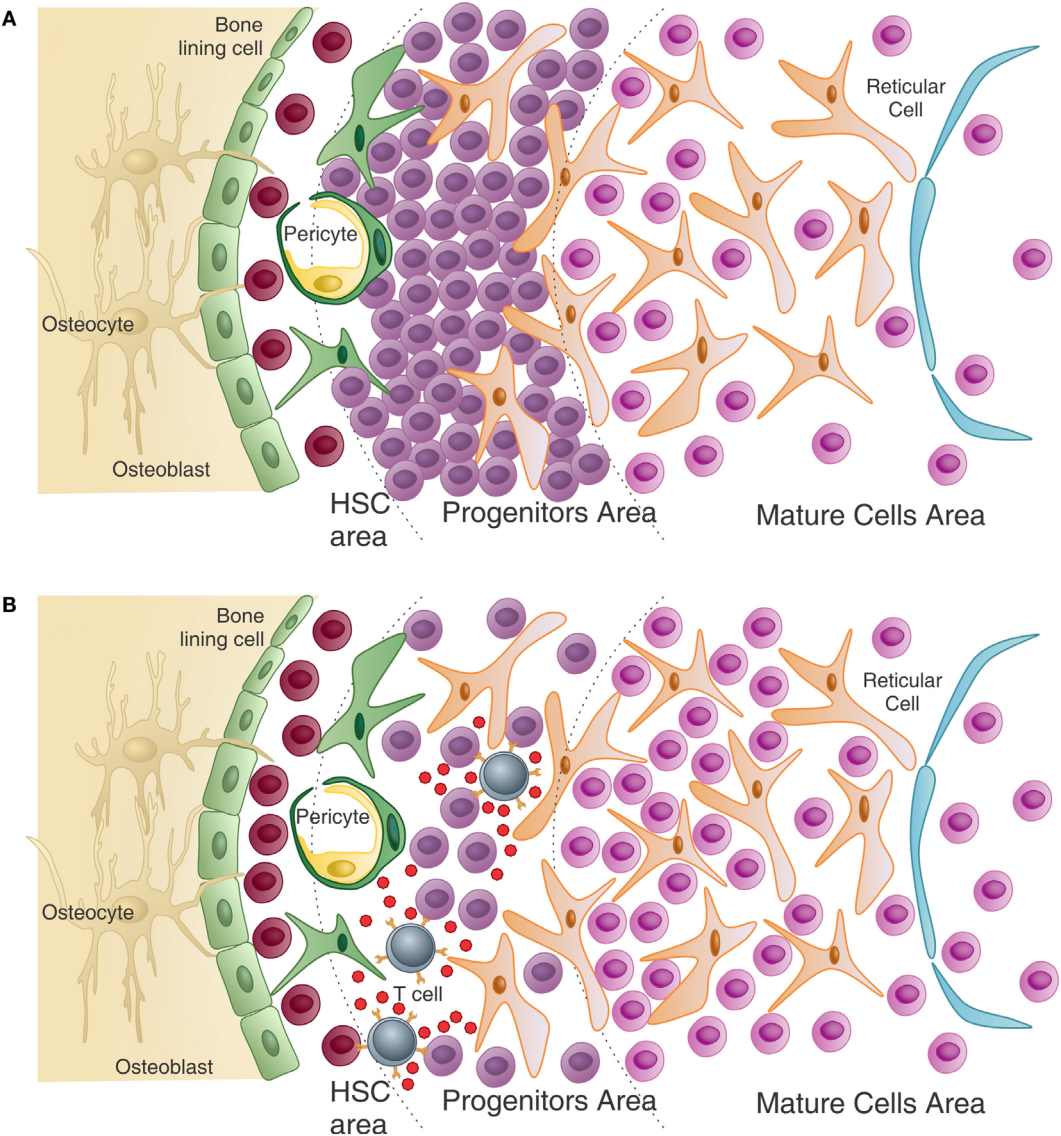
**T cells help normal hematopoiesis – (A) in the absence of T cells, or in the presence of non-activated T cells (not represented), immature progenitors cells, particularly of the myeloid lineage, accumulate in the bone marrow resulting in peripheral granulopenia**. **(B)** When activated T cells are present, they can see antigen in several hematopoietic cells and release growth factors, in return. These cytokines can directly and indirectly affect maturation of myeloid progenitors. Also, T cell cytokines modulate the stroma, which in turn can modulate HSC maintenance and myeloid differentiation. The number of HSC is also higher than in the T-less environment (adapted from Journal of Cellular Biochemistry, 2014, CordeiroSpinetti et al. with permission from John Wiley & Sons).

Using athymic mice, our data suggest that T cell interactions with hematopoietic precursor populations maintain hematopoiesis. These athymic mice exhibit granulocytopenia in the peripheral blood, as immature granulocytes accumulate in the BM (Figure 2A). Importantly, immature hematopoietic cells in the BM have no intrinsic defect that prevents their differentiation, since *in vitro* stimulation with growth factors induces their differentiation and maturation. Thymus graft or CD4^+^ T cells’ adoptive transfer, but not CD8^+^ T cells, rescues normal hematopoiesis and restores peripheral granulocytes counts. These CD4^+^ T lymphocytes present in the BM are predominantly activated and produce IL-4 and IFN-γ, even in the absence of any intentional external antigen stimulation (42, 81). However, if T cells are virgin, meaning they have yet to interact with their cognate antigen (as in TCR transgenic mice specific for an external antigen), peripheral granulopenia with accumulation of myeloid blasts in the BM is observed (42). “Normal” hematopoiesis is restored, however, if T cells are activated with their cognate antigen (Figure 2B). This work demonstrates that, among its functions, T cell cross talk with hematopoiesis plays a critical role in “normal” hematopoiesis.

One critical demonstration of this relationship comes from studies with hyper IgM immunodeficiency patients. These patients have a mutation in the CD40L gene, and their activated T cells do not assist B cells and macrophages in their activities, even in the presence of cognate antigen (82–84). However, these patients are also granulopenic, suggesting that CD40L is also important for hematopoiesis. In fact, CD40L engagement induces the production of Flt3L by different cell types and thrombopoietin by BM stromal cells (85). Moreover, human CD34^+^ hematopoietic progenitor cell are CD40 positive and when CD40L binds to it, proliferation and myeloid differentiation take place, mainly on the dendritic cell (DC) pathway (86).

### BM *In Situ* Signaling and Delivery of Cytokines: T Cells Bring the News

Studies on hematopoietic cells from the BM and the spleen of mice with diverse T helper cell polarization profiles provided clues on how T cells influence normal hematopoiesis. In mice with targeted deletion of STAT-4, which are biased to the Th2 phenotype, the number and cycling status of hematopoietic cells are significantly decreased compared to wild-type mice. By contrast, STAT-6 knockout mice, which are biased to Th1 response, demonstrate increased numbers of progenitors and cycling cells. This phenomenon is attributed to oncostatin M (OSM) secreted by Th1 T cells, since treatment of STAT-4-deficient mice with OSM recovers the number of cycling progenitors in the BM (87). From these observations, it becomes clear that activated BM T cells play an important role in hematopoiesis.

Further examples of T cells regulating hematopoiesis abound. For example Rådinger and colleagues (88, 89) reported that CD8^+^ T lymphocytes have a regulatory role in the control of eosinopoiesis in the BM. Transgenic mice overexpressing IL-5 in CD3^+^ cells and deficient in CD8^+^ cells show a significant decrease in BM eosinophils in steady state, what is not observed in the partner knockout mice to CD4^+^. In addition, adoptive transfer of naive CD8 T lymphocytes into immunodeficient mice (SCID-bg) restores normal levels of eosinophils in the BM. By contrast, in airway allergen challenge both T CD4 and CD8 subsets seem to be important in BM and bronchoalveolar lavage (BAL) eosinophilia (88, 89). Another example of hematopoiesis regulation by T cells comes from adoptive transfer of activated antigen-specific CD8 T lymphocytes, leading to increased numbers of multipotent and committed hematopoietic progenitor cells, especially myeloid lineage. These CD8 T cells secrete IFN-γ, which in turn acts on BM mesenchymal stromal cells, inducing these cells to release hematopoietic cytokines, especially IL-6. IL-6 modulates the expression of transcription factors essential to myeloid commitment and promotes accumulation of myeloid cells in the periphery (90).

Another molecule that implicates T lymphocytes in hematopoiesis is granulocyte macrophage colony-stimulating factor (GM-CSF). GM-CSF is a hematopoietic growth factor that stimulates proliferation of myeloid progenitor cells. It induces survival and activation of mature myeloid cells, and has a pro-inflammatory role in different diseases (91, 92). Interestingly, the hematopoietic system of GM-CSF knockout mice is normal (93). However, these mice when infected with *Mycobacterium avium* (94) or *Listeria monocytogenes* (95) have severe depletion of BM hematopoietic cell numbers, a deficient inflammatory response and are unable to increase their colony-forming cells in BM. This suggests that GM-CSF is essential for a hematopoietic response emergency, but not required for normal hematopoiesis. Interestingly, Th1 and Th17 lymphocytes are able to produce and secrete GM-CSF when activated by different stimuli (96–99). Recently, GM-CSF producing T cells (Th-GM) were reported. Generation of GM-CSF^+^/IL3^+^ CD4 lymphocytes is mediated by IL-7/STAT-5 signaling, and Th-GMs do not express the transcription factors T-bet and ROR-γt, suggesting that these cells belong to a distinct subset of Th cells (100, 101).

The effects of IL-17 in hematopoiesis appear to be largely indirect through the induction of cytokine secretion, including GM-CSF, G-CSF, SCF, NO, IL-6, prostaglandins, and other chemokines (102–104). BM stromal cells are the main target of IL-17 as stromal and MSCs, express a high level of IL-17RA (105, 106).

### Treg Inhibits Differentiation and Preserves the HSC Pool

Recently, Treg lymphocytes involvement in hematopoiesis was investigated. The frequency of FoxP3 Treg cells in CD4 T cells is higher in the BM (around 20%) than that in the spleen and lymph nodes (10–15%) (107, 108). Treg cells suppress the function of CD4 and CD8 T cells. In a murine model of allogeneic hematopoietic stem cell transplantation (HSCT) into non-irradiated recipients, Tregs co-localized with HSCTs and accumulated close to endosteum surface in the calvaria and trabecular BM. When Treg cells are depleted, a 70% reduction in the number of surviving donor cells, with concomitant increase in levels of TNF-α and IL-4 producing CD4^+^FoxP3^−^ and CD8^+^ T BM cells is observed. These data suggest that Tregs provide an immune privilege mechanism that enables transplanted allo-HSPCs to escape allogeneic rejection in the BM (107). However, using a direct approach, Urbieta and colleagues (109) demonstrated that active Tregs are able to prevent the differentiation of IL-3/SCF colony-forming units (CFU) progenitor cells *in vitro* and this inhibition depends upon T cell antigen recognition and TGF-β, but not on FasL and perforin. Moreover, in syngeneic HSCT, Treg depletion either in host mice or in donor cell population increases splenic CFU-IL3 after transplantation indicating that donor and host Tregs can modulate hematopoiesis. In line with these results, addition of Tregs to T-cell-depleted transplants diminishes splenic and BM-derived CFU and peripheral neutrophil counts (109). It seems that Tregs indeed protect immature progenitors from differentiating and, most probably, help to keep the stem cell pool, although this deserves a more detailed investigation.

### Peripheral Demand and Central Hematopoiesis Holding Hands

During infections and inflammatory conditions, immune effector cells, especially neutrophils, are highly demanded and rapidly consumed in peripheral tissues. Although adaptive immune cells (T and B lymphocytes) respond to efficient antigen presentation with activation and proliferation, innate immune cells need to be replenished to maintain the progenitor cells pool. The hematopoietic response to this need is named demand-adapted hematopoiesis or emergency hematopoiesis.

T lymphocytes fit well with the definition of “messengers from the periphery to the BM” (Figure 2). The beauty of the system is that the pleiotropic effect of T cell interaction with multiple systems coordinates the immune response “class” in the adaptive as well as in the innate compartment. Th2 responses are the example prototype, with IL-4 being the master cytokine orchestrating the immune response. In the periphery, Th2 cells help B cells with a consequent switch in immunoglobulin class to IgE. In the BM, IL-4 induces mast cell differentiation. IgE has no function alone and works in pair with mast cells, made possible by the stimulation of IgE production by B cells and mast cells differentiation from BM progenitors, in a coordinated manner. Similar to this, Th1 responses induce IgG2a class switch in B cells and activate macrophages through IFN-γ, which inhibits osteoclast (OC) differentiation favoring the monocyte differentiation pathway (110). IgG2a is a good opsonin to which macrophages have high-affinity receptors. Th1 activity in the BM increases differentiation of monocytes, which have high-affinity receptors for IgG2a, and the immunoglobulin isotype is also stimulated by Th1 cells. In parallel to what was described above for Th2 cells, Th1 cells coordinate hematopoietic activity with adaptive immunity, with the immunoglobulin class switch improving innate immunity. Less obvious is the Th17 response, which is closely related to indirect actions, but signals on epithelial and stromal cells licensing those for production of cytokines, which will stimulate granulopoiesis and neutrophil migration to the damaged tissue. These cells are short lived and highly demanded, especially in the first phase of the response, needing to be replaced and produced in accelerated rates. This can be achieved after the Th17-specific cell engages its TCR on the surface of an antigen-presenting cell (APC) in the BM (42, 111, 112). Th17 cells secrete IL-17 and GM-CSF and stimulate BM stromal cells to produce G-CSF, modifying hematopoiesis based on antigen-specific peripheral demand.

There are still many points to be considered and investigated in the relationship between T lymphocytes and the hematopoietic system. However, the data gathered here provide strong evidence that this is not a simple and static relationship; it is modulated according to BM microenvironment, as well as by changes in the external environment sensed with the help of activated T cells.

## T Cell and the Bone: Regulating the Bone Remodeling System

### Bone Remodeling: The Players

The skeletal system renews itself completely every 18 years, indicating its dynamic metabolism. Subendosteal perivascular cells serve as source for new osteoprogenitors in the BM cavity (68, 113, 114). As bone remodels, several factors released from the matrix after OC-mediated bone resorption will recruit bone-lining cells to become activate osteoblasts (113, 115). Osteoblasts will then deposit new bone matrix and further mineralize it. During bone deposition, a few osteoblasts get trapped in the matrix and become fully differentiated osteogenic cells, turning into osteocytes (113, 115).

Osteoclasts, on the other hand, derive from the hematopoietic lineage. OCs are bone resident cells involved in physiological and pathological remodeling of bone tissue. Mature OCs are tartrate-resistant acid phosphatase (TRAP), calcitonin receptor, cathepsin K, αβ3-integrin, and adenosine triphosphate-dependent proton pump subunits positive, multinucleated giant cells that are generated from the fusion of precursors derived from the monocyte/macrophage lineage (116). Mature OCs polarize and adhere to the bone matrix, acidify the bone surface, release osteolytic enzymes, and resorb bone. Two factors appear as crucial for OC differentiation: macrophage colony-stimulating factor (M-CSF), which induces monocyte differentiation, and RANKL, which is essential for stimulating pre-OC differentiation and its merge into multinucleated cells. Both factors are believed to derive mainly from osteoprogenitor cells and pre-osteoblast. Osteoblasts, on the other hand, produce and secrete osteoprotegerin (OPG) that is a RANK decoy receptor and inhibits OC formation and bone resorption (116).

**Figure 3:**
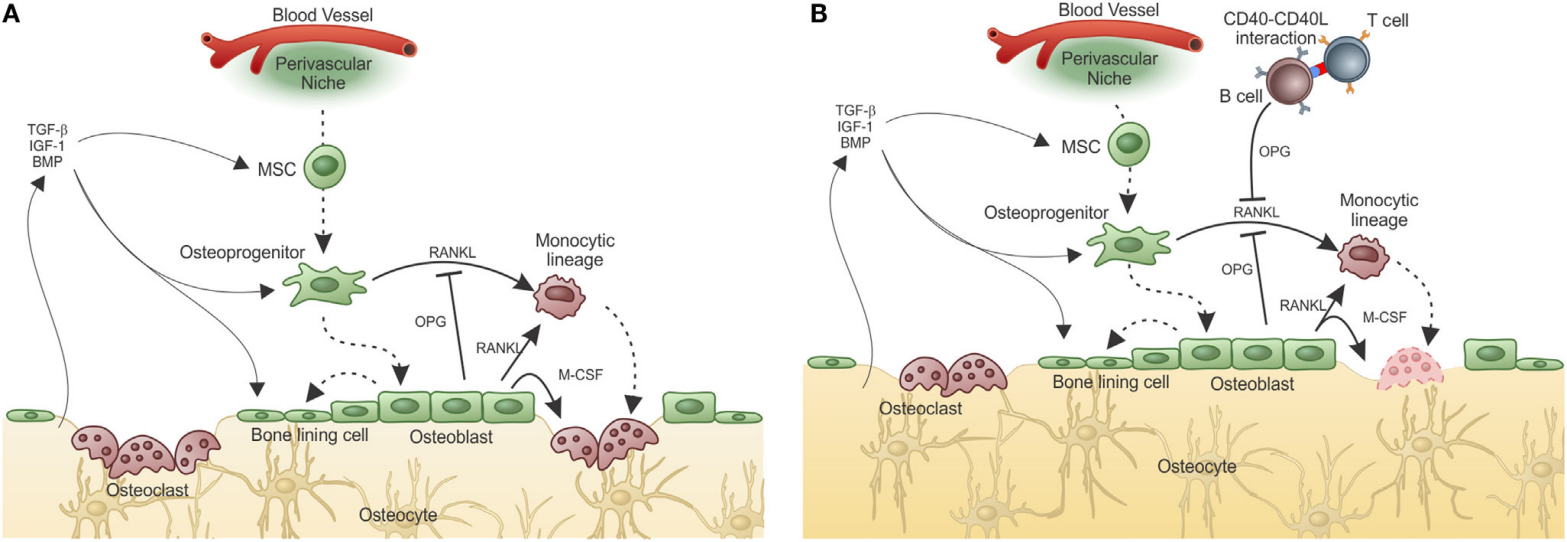
**Adaptive immunity participates in physiological bone remodeling – (A) bone remodeling relies on osteoclasts (OC), which in physiological situations differentiate from OC precursors of the monocytic lineage after signaling through RANK in its surface and RANKL produced by osteoprogenitor cells**. As the bone is resorbed, growth factors and other molecules stored in the extracellular matrix are released, including TGF-β that stimulates osteoblastogenesis from MSC, which reside in the perivascular niche (as in the representation) or in the endosteal niche (not represented). As a consequence, new osteoblasts are formed resulting in bone formation. OPG, a decoy receptor for RANKL, is produced by osteoblasts and participate modulating the bone remodeling system. **(B)** When activated T cells are present, they activate B cells in a CD40–CD40L-dependent manner with OPG production in large quantities by B cells. This leads to bone accumulation as the RANK–RANKL axis is disrupted by the high amounts of OPG produced by B cells. This is what defines the “normal” bone mass in physiological situation, in SPF condition. Dashed lines represent cellular differentiation; filled line represents cytokine action.

Under physiological conditions, the balance between bone formation and resorption is finely tuned and can be disrupted under non-homeostatic conditions (Figure 3A).

### RANK–RANKL–OPG Axis: Linking the Immune and Skeletal System

RANKL (also known as ODF, TRANCE, OPGL, and TNFSF11) and its receptor RANK (also known as TRANCE-R and TNFRSF11A) central players in bone metabolism (117) were first described as molecules expressed in T cells and DCs. OPG, the soluble receptor/antagonist for RANKL, initially characterized as an inhibitor factor of OC development and bone resorption, expressed by osteoblasts (116, 118), and B cells (119, 120), has also been described as a crucial regulator of B cell maturation and development of antibody responses (121). In fact, the RANK/RANKL system also controls lymph node organogenesis and development of thymic medullary epithelial cells and lymphocytes (122–124). The RANK–RANKL–OPG axis also has important functions in other systems, such as the lactating breast, and in the thermo-regulatory system (125). Regulation of tumor cell migration to the bone as well as primary mammary tumor growth had also been reported and relate to physiological activities of these molecules (126).

The role of the adaptive immune system in what is known as the equilibrium between bone consumption and deposition in healthy animals was addressed by Li et al. (119). They showed that acquisition of normal bone mass depends on the presence of OPG produced by mature activated B cells. However, in the absence of T cells, B cells do not produce OPG and bone remodeling is unbalanced toward bone consumption due to lack of the inhibitory OPG–RANKL loop. Moreover, OPG production by B cells was dependent upon CD40L from activated T cells, showing that T–B interaction is required for “normal” bone density maintenance (Figure 3B).

Evidence related to hormonal (estrogen) regulation of TNF-α production by T cells have also been published, and data link adaptive immunity to bone activity (127). However, the results vary depending on the model used (128). In the presence of B cells, but in the absence of T cells, bone mass decreases after ovariectomy, in accordance to Li et al. (119). In this case, B cells are present, but are not activated by T cells. However, if both, B and T cells are absent, as in RAG-deficient mice, bone mass increases after ovariectomy making any conclusion about the role of estrogen in regulation of T cell activity over the bone difficult at the present moment.

Apart from hormones, another important evidence of T cell participation in physiological bone remodeling comes from studies in germ-free (GF) mice (127, 129). These animals have lower numbers of T cells in the BM and these are not activated. Also, GF mice have low frequency of OCs and are osteopetrotic. Coincidentally, colonization with commensal microbiota reverses the osteopetrotic phenotype and restores normal numbers of activated T cells, as well as of OC precursors (130) These data are in accordance with the view that in steady-state healthy conditions, recognition of antigen by T cells tunes the bone remodeling system.

T-cell-derived RANKL can contribute to bone loss in some pathological conditions, as it was first proposed by Kong and colleagues in a model of inflammatory bone disease (131). Later studies showing reversal of RANKL-dependent osteopetrosis by hyper-expression of RANKL in T and B cells (132) reinforced the interplay between adaptive immune cell-derived RANKL and bone metabolism. On the other hand, Sato et al. better described the involvement of T-cell-derived IL-17A and interaction with RANKL in synoviocyte and macrophages in the inflamed joint (133, 134).

Generally, the cross talk between both systems has been studied in models of arthritis, in which the action of T cells in an inflamed joint stimulates osteoclastogenesis and bone resorption through the indirect action of T-cell-derived IL-17A and RANKL (134–137). However, in the arthritis models, T cells reach the bone through the articular cavity after an inflammatory injury, which destroys the cartilage and exposes the periosteal bone surface.

**Figure 4:**
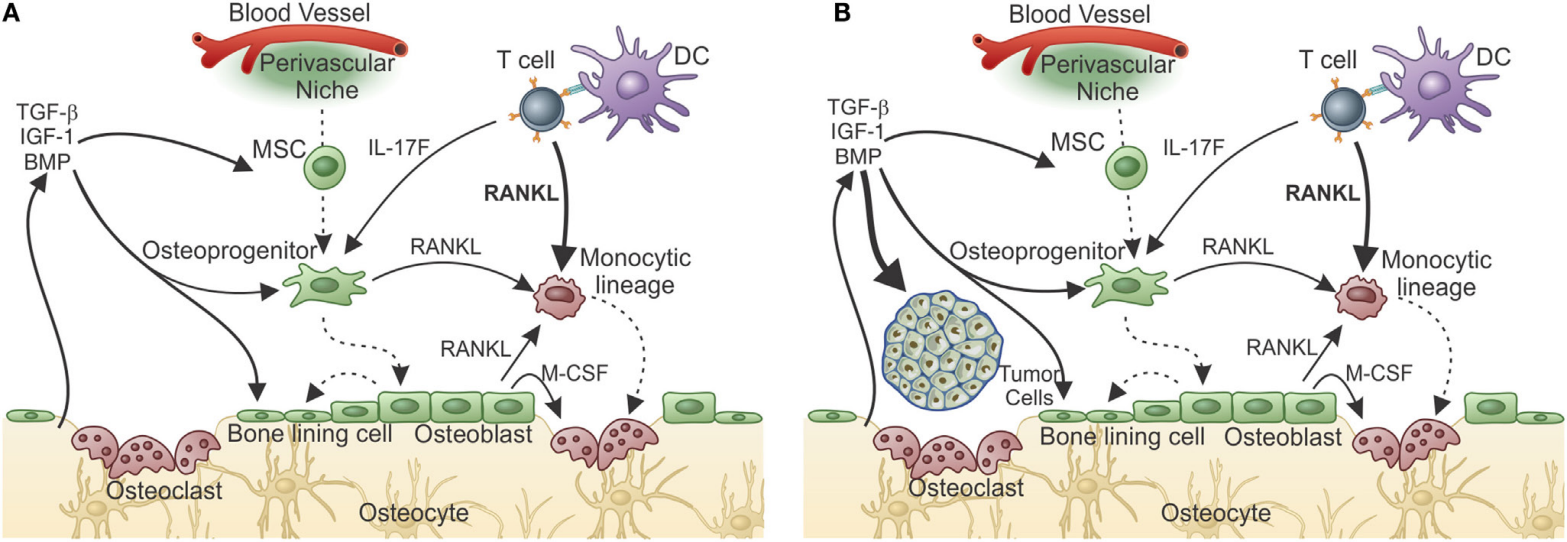
**T-cell-derived RANKL prepares the bone pre-metastatic niche**. **(A)** After responding T cells meet tumor antigen and get activated, they migrate to the bone marrow where, after encountering antigen, they release cytokines. In the case of a metastatic tumor, responding T cells produce IL-17 and RANKL. Although IL-17F from T cells can indirectly influences osteoclastogenesis, T-cell-derived RANKL is the central cytokine stimulating osteoclastogenesis and bone consumption resulting in pre-metastatic niche organization with growth factor release from the resorbed matrix. **(B)** The growth factor-rich environment will nourishes the arriving tumor cells allowing metastatic colonization. In the absence of T-cell-derived RANKL, metastatization into the bone does not take place.

In contrast to these mechanisms observed in arthritis, in other conditions, the T cells normally present inside the BM and in contact with the bone might contribute to pathological bone remodeling. In fact, we have recently shown that RANKL production by tumor-primed BM CD4^+^ T cells induces pre-metastatic osteolytic bone disease that licenses metastatic colonization (138). We reached this conclusion by showing that the 4T1 breast tumor induces production of pro-osteoclastogenic cytokines, IL-17F, and RANKL by BM CD4^+^ T cells, leading to OC formation and activation *in vitro* and *in vivo*. Surprisingly, osteolytic disease was observed even before tumor cells colonized the bone and was induced by T cell transfer, showing that T cells prepare the metastatic niche for further establishment of tumor cells (Figure 4A). Thus, the T cell mediated pre-metastatic osteolytic disease adds an extra step to the vicious cycle hypothesis, which established that bone colonization by tumor cells culminate in the release of growth factors from the bone matrix as a consequence of OC activity (Figure 4B) (139, 140).

### Osteoclasts and Dendritic Cells: Modulators of the Bone Remodeling Gear

Interactions between monocytes/macrophages and OCs has long been studied and was appreciated in the pioneering work of Horton et al. in the early 1970s (141). The set of soluble factors secreted from antigen-stimulated peripheral blood mononuclear cells was initially described as an “OC-activating factor,” which subsequently was identified as predominantly interleukin-1 (IL-1) and TNF (142). In addition, numerous macrophage-derived cytokines influence OC differentiation and/or function, such as M-CSF, which are essential for the development and survival of the monocyte/macrophage lineage as well as OC differentiation (136).

One important cell type also derived from monocytic progenitors and precursors in the BM is the DC, the most potent APC, responsible for activating naive T cells, and orchestrating the immune responses (143). Since DCs are derived from the same monocyte/macrophage OC precursor lineage, it is not surprising that splenic immature DCs can be developed into OCs (OC–DC), when cultured with osteoclastogenic factors M-CSF and RANKL (144–146).

Accumulated evidence indicates that mature OCs can function as APCs to both CD4^+^ and CD8^+^ T cells *in vitro*. They express MHC I and II, CD80, CD86, CD40, IL-10, TGF-β, IL-6, and TNF-α, all molecules that act on T cell activation and class switch (147). It has been suggested that the monocyte population used to differentiate OCs carry contaminant DCs or monocyte themselves, which could explain the antigen-presenting capabilities of OCs. OCs can in fact be derived from immature DCs *in vivo* and *in vitro* (148–150), although the antigen-presenting capability of these OC–DCs had never been addressed.

Circulating DCs are normally recruited to inflamed tissues (149, 151) and IL-23-producing DCs play an indirect role in osteoclastogenesis by stimulating T cell expression of RANKL and IL-1, IL-6, IL-17, and TNF-α that augment the release of TRAP and cathepsin K by resident OCs (149). A still-open question is whether the same growth factors that control normal osteoclastogenesis are also involved in generating OCs in response to inflammation with potent DC activity. Participation of OC–DC in bone remodeling was suggested in Langerhans cell histiocytosis (LCH). LCH is a multi-symptom disease with associated bone resorption and granuloma formation, and soft tissue inflammatory lesions (152). Bone pathology appears to rely on DCs and monocytes, which produce high levels of IL-17A and induce DC–OC differentiation (150, 153). Also it was shown that DCs could induce osteolysis when transferred to regular or osteopetrotic mice (154). Osteolytic activity *in vivo* was inhibited with OPG and bisphosphonates, implying a role for RANKL in the pathogenic DC–OC pathway (154).

**Figure 5:**
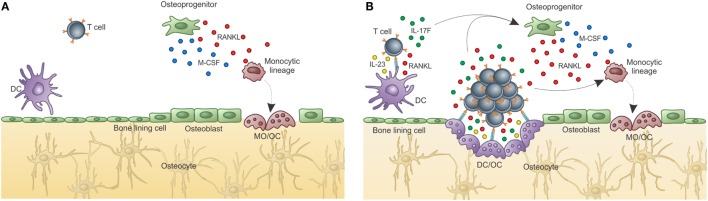
**Osteoclasts are optimal antigen-presenting cells when derived from DCs, but not from BM progenitors**. Osteoclasts are usually derived from bone marrow progenitors or monocytic precursors (OC), but can also derive from dendritic cells under certain conditions (OC–DC). **(A)** Classical “constitutive” OC. OC are not good antigen presenters, cannot activate T cells, and do not maintain T cell activation in the BM. Thus, OCs do not contribute to T cell activation in the BM. **(B)** Activation induced OC–DC. Under the influence of RANKL among other cytokines, OC–DCs are induced. If OC–DCs are as good as fresh DCs in presenting antigen to T cells and if they provide IL-23, a positive loop will be built, which can amplify the osteoclastogenic potential of T cell stimulation. In this context, the bone-resorbing activity of both DC–OC and classical OC will result in decreased bone mass (represented by the light color of the matrix). Whether both OCs dwells in the same resorbing pit, or are segregated into separate subniches, as represented in B, is unknown.

Since it was shown that DC can prime T cells inside the BM (155), it can be reasoned that a DC can acquire OC characteristics while keeping its antigen-presenting capabilities. If this is true, then DCs can work as messengers from the periphery to the BM where they maintain T cell activation/memory and interfere with bone metabolism, in addition to participating in hematopoiesis (Figure 5).

## Final Remarks: Tuning the BM Multisystem

The skeletal system provides vital functions, as mechanical structure, movement, protection, calcium storage, endocrine regulation, and support for the whole hematopoietic system. Blood cell production is a very finely controlled process, which depends upon stromal cells and their factors, and cell intrinsic conditions. In the BM cavity, HSCs and progenitors reside close to the bone surfaces, which suggests that the two systems are integrated.

Bone remodeling’s influence over hematopoiesis is not clear, although there are reports in the literature, indicating that osteoblast activity increases the number of primitive progenitors (24, 31, 32, 38) and also that increased numbers of OCs might induce HSC mobilization (28, 32, 115, 156–158). It is possible that OC mobilization and activation together disrupt HSC niches as the stem cells reside close to the bone surface and in close contact to osteoblasts. As both OCs and OBs are involved in bone remodeling, stem cell niches are disrupted and the immature progenitors leave the quiescent niche and are enabled to enter the proliferation and differentiation pathway. T cells participate on maintaining or disrupting the balance of both systems depending on their activity. In the case of pro-osteolytic activity, when T cells get inside the BM, they can see antigen on IL-23^+^ DCs, keeping their Th17 profile, producing RANKL and inducing osteolysis. This will disrupt hematopoiesis through osteoclastogenesis stimulation, which disturbs the HSC niche. At the same time, T cells can produce IL-17A, F, and G-CSF (98, 99), which indirectly stimulates neutrophil maturation, providing “help” for the immature myeloid progenitors recently released from the stem cell niche.

**Figure 6:**
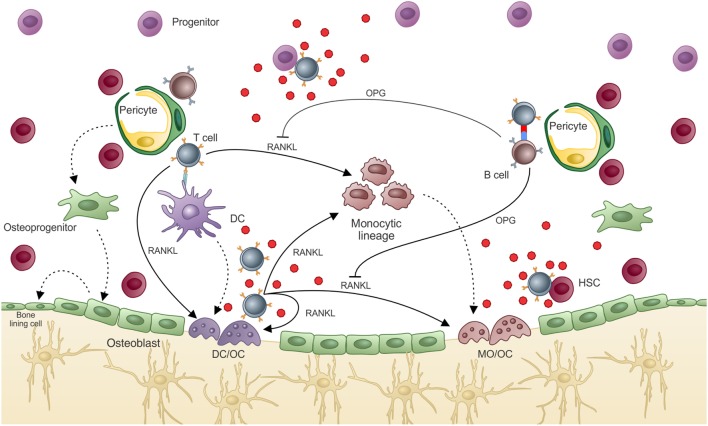
**T cell regulation of bone marrow environment**. T cell participation in the BM environment promotes what we know as “normal hematopoiesis” and “normal bone mass.” In hematopoiesis, T cell release of hematopoietic cytokines after activation allows terminal differentiation of hematopoietic progenitors/precursors, which will compose the “normal” peripheral pool. “Normal” bone density is also dependent on T cell activation, which in a normal state activates B cells to produce OPG, balancing bone resorption by OCs and mineral deposition by OBs. If T cells are highly activated or are turned into an osteoclastogenic phenotype, excessive bone resorption can lead to osteolytic disease. Moreover, some of the cytokines produced by T cells that can interfere with the bone remodeling system can also affect hematopoiesis, such as IL-17F.

In summary, we believe that T cells pave the road that unravels the partnership of the three systems (i.e., immunity, hematopoiesis, bone homeostasis) by acting as messengers from the periphery to the bone cavity. It is clear that inside the BM, T cells bring information from the periphery. They produce cytokines and express membrane molecules, which will act in an antigen-specific manner. Although it remains unclear as to the need for a cognate antigen inside the BM, it is clear that only after *in vivo* priming can BM T cells acquire an activated phenotype. Whether or not T cell regulation of bone homeostasis requires recognition of self-antigens by DCs or OCs inside the BM, or if antigen-loaded APCs do the job or both, is still not clear. However, it is clear that if BM T cells recognize antigen (self or not) on APCs, they will exert their effector functions “helping” the bone remodeling and hematopoietic system. The final effect on hematopoiesis and bone remodeling depends on the class of the T cell response (159), identified by the cytokine package produced and released by the T cell subset (Figure 6).

## Author Contributions

ABo, AM, TG-S discussed the literature anrd wrote about T cells inside the bone marrow; EC-S and ABa discussed and wrote about the bone marrow organization and its niches. RG prepared the illustrations, discussed and reviewd the paper. ABo and ABa revised and put together the niches, in the bone marrow and the adaptive immune system.

## Conflict of Interest Statement

The authors declare that the research was conducted in the absence of any commercial or financial relationships that could be construed as a potential conflict of interest.
